# Active glycolytic metabolism in CD133(+) hepatocellular cancer stem cells: regulation by MIR-122

**DOI:** 10.18632/oncotarget.5812

**Published:** 2015-10-19

**Authors:** Kyoungsub Song, Hyunjoo Kwon, Chang Han, Jinqiang Zhang, Srikanta Dash, Kyu Lim, Tong Wu

**Affiliations:** ^1^ Department of Pathology and Laboratory Medicine, Tulane University School of Medicine, New Orleans, LA, USA; ^2^ Department of Biochemistry, College of Medicine, Cancer Research Institute and Infection Signaling Network Research Center, Chungnam National University, Daejeon, Korea

**Keywords:** CD133, cancer stem cells, glycolysis, miR-122

## Abstract

Although altered metabolic pathway is an important diagnostic maker and therapeutic target in cancer, it is poorly understood in cancer stem cells (CSCs). Here we show that the CD133 (+) hepatocellular CSCs have distinct metabolic properties, characterized by more active glycolysis over oxidative phosphorylation, compared to the CD133 (−) cells. Inhibition of PDK4 and LDHA markedly suppresses CD133 (+) stemness characteristics and overcome resistance to sorafenib (current chemotherapeutic agent for hepatocellular cancer). Addition of glucose or lactate to CD133 (−) cells promotes CSC phenotypes, as evidenced by increased CD133 (+) cell population, elevated stemness gene expression and enhanced spheroid formation. Furthermore, the liver-specific miRNA, miR-122, inhibits CSC phenotypes by regulating glycolysis through targeting PDK4. Our findings suggest that enhanced glycolysis is associated with CD133 (+) stem-like characteristics and that metabolic reprogramming through miR-122 or PDK4 may represent a novel therapeutic approach for the treatment of hepatocellular cancer.

## INTRODUCTION

Tumor cells exhibit phenotypic and functional heterogeneity as a consequence of genetic, epigenetic change and environmental differences [1]. Cancer stem cells (CSCs), sometimes referred to as tumor initiating cells (TICs), have been postulated as a small population of cells that drive tumor growth and contribute to heterogeneity in various cancers [2]. They express specific cell-surface markers such as CD133, CD44 and EpCAM; these markers can be used to isolate CSC population from primary tumors and established cancer cell lines [3]. These cells are known to be responsible for driving tumorigenesis and conferring resistance to chemotherapeutic agents [4].

Hepatocellular carcinoma (HCC) is the most common types of liver cancer and the third leading cause of cancer-related death [5]. Recent studies have identified liver CSCs population using cell surface markers including CD133, EpCAM and CD90, and demonstrated their self-renewal abilities, tumorigenic potential and chemoresistances [6–9]. However, to date, the environmental, cellular and molecular factors that regulate liver cancer stem cells and their mechanisms of actions remain incompletely understood.

Altered cellular metabolism is a major feature of cancer. The Warburg effect, characterized by elevated glycolysis and lactate production in the presence of oxygen, is a common metabolic alteration of cancer cells [10]. Likewise, many types of stem cells possess aerobic glycolysis, and these predominant glycolytic phenotype is associated with stem cell function [11]. In this context, predominant glycolysis is common metabolic properties both in cancer cells and stem cells. Although several recent studies have reported the metabolic phenotypes of CSCs in several cancers including breast, colon and brain cancer [12–14], the metabolic properties of CSCs are controversial, and to date there are no studies to investigate the metabolic phenotypes and their mechanism in liver CSCs.

Accumulating evidence suggests that miRNA plays an important role in carcinogenesis and tumor progression [15]. miR-122 is a liver-specific miRNA that constitutes approximately 70% of total miRNAs in the adult liver; its expression is developmentally regulated [16]. miR-122 regulates hepatic lipid and cholesterol metabolism in the liver, and also has tumor suppressive role in HCC. Recently, several studies have shown that germ line and liver specific miR-122 knockout mice (miR-122 KO and miR-122 LKO) spontaneously developed hepatocellular carcinoma [17, 18]. miR-122^−/−^ tumor cells showed elevated expression of oncofetal genes (Afp, Igf2 and Src) and CSCs surface markers (CD133 and EpCAM) [18]. However, it remains unknown whether and how miR-122 impacts liver CSCs.

The aim of this study was to determine the metabolic profiles, especially glucose metabolism, between CD133 (+) liver CSCs and CD133 (−) cells. We report herein that CD133 (+) CSCs are highly glycolytic and that their stemness characteristics are significantly reduced when glycolysis is inhibited. We further show that extracellular glucose and lactate are important environmental factors to maintain stemness characteristics of CSCs (as reflected by stemness genes expression and spheroid formation). Additionally, our results demonstrate an important role of miR-122 for regulation of glycolysis and stemness characters. While miR-122 is markedly down-regulated in CD133 (+) cells, ectopic expression of miR-122 inhibits glycolysis (via targeting PDK4) leading to decreased stemness gene expression and reduced spheroid formation. These results provide important evidence for glycolysis in liver CSCs maintenance and suggest that targeting glycolytic pathway through miR-122 may represent a potential therapeutic strategy to eradicate liver CSCs.

## RESULTS

### Characterization of CD133 (+) human HCC cells

**Figure 1:**
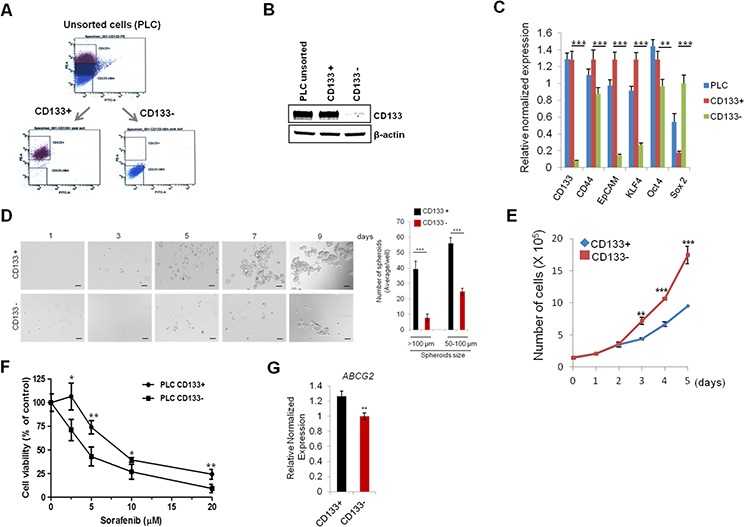
Characterization of CD133+ PLC/PRF/5 human HCC cells **A.** Flow cytometry panels show the distribution of CD133+ cells and CD133− cells. The top 20% most bright stained or bottom 10% most dimly stained cells were selected as positive or negative populations. **B.** Western blotting for CD133 in unsorted, CD133+, and CD133− PLC/PRF/5 cells. **C.** qRT-PCR was performed for CSC-associated genes. **D.** Sphere forming efficiency of CD133+ and CD133− PLC/PRF/5 cells. The cells were plated at low density (1,000 cells/well) on low attachment plates and sphere formation was imaged by indicated time points. The number and size of spheroid in each well were determined under light microscopy (right panel). **E.** Cell growth rate of CD133+ and CD133− cells. **F.** CD133+ PLC/PRF/5 cells were relatively resistant to sorafenib. The cells were treated with various concentration of sorafenib for 48 hrs and the cell viability was determined by trypan blue exclusion assay. **G.** qRT-PCR analysis for ABCG2 in CD133+ and CD133− cells. All experiments were performed at least three times independently and the data shown are mean ± S.D. from three technical replicates of the experiments. *p < 0.05; **p < 0.01; ***p < 0.001 (two-tailed Student's *t*-test).

Previous studies have shown that CD133 is a potential marker for cancer stem cells in several solid tumors including breast, brain and liver cancers [19]. In the current study, we used flow cytometry to isolate CD133 (+) and CD133 (−) cell populations from PLC/PRF/5 human hepatocellular cancer cell line by using PE-conjugated CD133 antibody (Figure 1A); the levels of CD133 were confirmed by Western blotting (Figure 1B). We then measured the expression of several stemness genes and other stem cell surface markers by q-RT-PCR. As shown in Figure 1C, CD133 (+) cells showed increased expression of stemness genes (KLF4 and Oct4) and other cell stem cell surface markers (CD44 and EpCAM). Given that cancer stem cells are known to grow as three-dimensional cellular aggregates in suspension (termed “spheroid”) [20], we measured the size and number of spheroids in our system. As shown in Figure 1D, the CD133 (+) cells formed more and larger spheroids compared to the CD133 (−) cells. To evaluate the proliferation rate of CD133 (+) and CD133 (−) cells, the sorted cells were cultured and the number of cells were counted at indicated time points. As shown in Figure 1E, CD133 (+) cells showed lower proliferate rate than CD133 (−) cells. The latter findings are consistent with the documented low cell proliferation in other cancer stem cells [21–23].

Accumulating evidence shows that CSCs are more resistance to conventional therapies than non-CSCs cells [24]. In HCC, sorafenib is the first and only FDA-approved therapeutic agent for systematic treatment of unresectable HCC [25]. However, sorafenib resistance is a significant clinical problem and the mechanism for sorafenib resistance remains incompletely understood. To test whether cancer stem cell characteristics may influence sorafenib resistance, CD133 (+) and (−) cells were treated with various concentrations of sorafenib (2.5 – 20 μM) for 48 hrs. As shown in Figure 1F, CD133 (+) cells were more resistant to sorafenib-induced cell death compared to CD133 (−) cells (growth inhibition rate, 26% *vs* 57% at sorafenib 5 μM, respectively). Interestingly, the CD133 (+) cells exhibited an increased expression of ABCG2, a member of the ATP-Binding Cassette transporters family, which is known to be implicated in drug-resistance (Figure 1G). Collectively, our data showed that the CD133 (+) cells have CSC phenotypes and are more resistant to sorafenib treatment. The sorafenib-resistant phenotype of CD133+ cells may relate to their slow growing property and their high expression of ABCG2.

### CD133 (+) CSCs are more glycolytic than CD133 (−) cells

**Figure 2:**
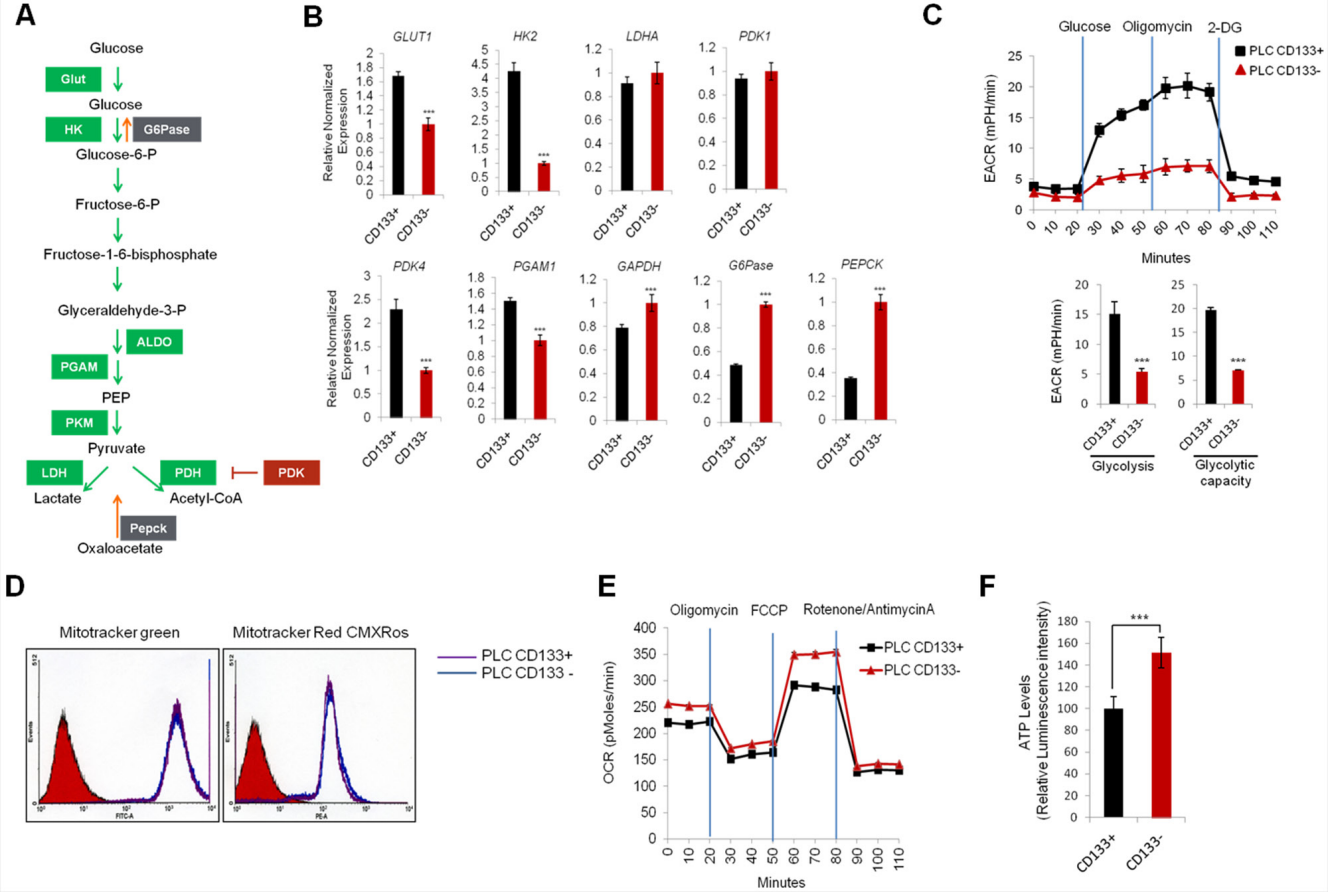
Glycolytic metabolism differences between CD133+ and CD133− PLC/PRF/5 cells **A.** Schematic representation of glycolytic pathway **B.** qRT-PCR analysis of glycolytic and gluconeogenetic gene expression. Glycolytic genes (Glut1, HK2, PDK4) were significantly upregulated and gluconeogenetic genes (G6Pase, Pepck) were downregulated in CD133+ cells compared to CD133− cells (***P < 0.001). **C.** Real-time measurement of extracellular acidification rate (ECAR) showed that CD133+ cells were more glycolytic than CD133− cells. The cells (35,000 cells/well) were seeded 24 hr prior to the assay. ECAR was measured after sequential incubation with 10 mM glucose, 2.5 μM oligomycin, 100 mM 2-DG. **D.** FACS analysis of CD133+ and CD133− cells stained with mitotracker green and mitotracker red CMXROS. **E.** Real-time measurement of oxygen consumption rate (OCR) in CD133+ and CD133− cells. OCR was measured after sequential incubation with 2 μM oligomycin, 2 μM FCCP and 0.5 μM Rotenone/antimycin A. **F.** Cellular ATP level was measured by luminescence microplate reader with ATPlite assay kit. Results were normalized to cellular protein concentrations. All experiments were performed at least three times independently and the data shown are mean ± S.D. from three technical replicates of the experiments. ***p < 0.001 (two-tailed Student's *t*-test).

To evaluate the metabolic characteristics of CD133 (+) cells, we performed qRT-PCR analysis to measure the expression of several metabolic enzymes that are implicated in glycolysis and gluconegogenesis (a schematic diagram of the glycolytic pathway is shown in Figure 2A). We observed that the CD133 (+) cells had increased expression of glycolytic enzymes (Glut1, HK2, PDK4 and PGAM1) and decreased expression of gluconeogenetic enzymes (G6Pase and Pepck) (Figure 2B). To further document the glycolytic capacity of CD133 (+) and CD133 (−) cells, we measured extracellular acidification rate (ECAR) using Seahorse XF24 Extracellular Flux analyzer. As shown in Figure 2C, the ECAR was significantly higher in CD133 (+) cells compared to CD133 (−) cells, which is consistent with the qRT-PCR data. We next measured mitochondrial mass and membrane potential by staining with Mito Tracker green and Mito Tracker red CMXRos. Our data showed no significant difference in mitochondria mass and membrane potential between CD133 (+) cells and CD133 (−) cells (Figure 2D). To further determine mitochondrial functions, we measured oxygen consumption rate (OCR). We observed that basal and maximal OCRs were all higher in CD133 (−) cells compared to CD133 (+) cells (Figure 2E). These results suggest that CD133 (+) cells possess more glycolytic phenotypes and less mitochondrial respiration than CD133 (−) cells. Furthermore, the intracellular ATP level was lower in CD133 (+) cells compared to CD133 (−) cells, which is in accordance with less ATP production by mitochondrial oxidative phosphorylation (Figure 2F).

### Glycolytic reprogramming inhibits CD133 (+) cell growth and stemness characteristics

**Figure 3:**
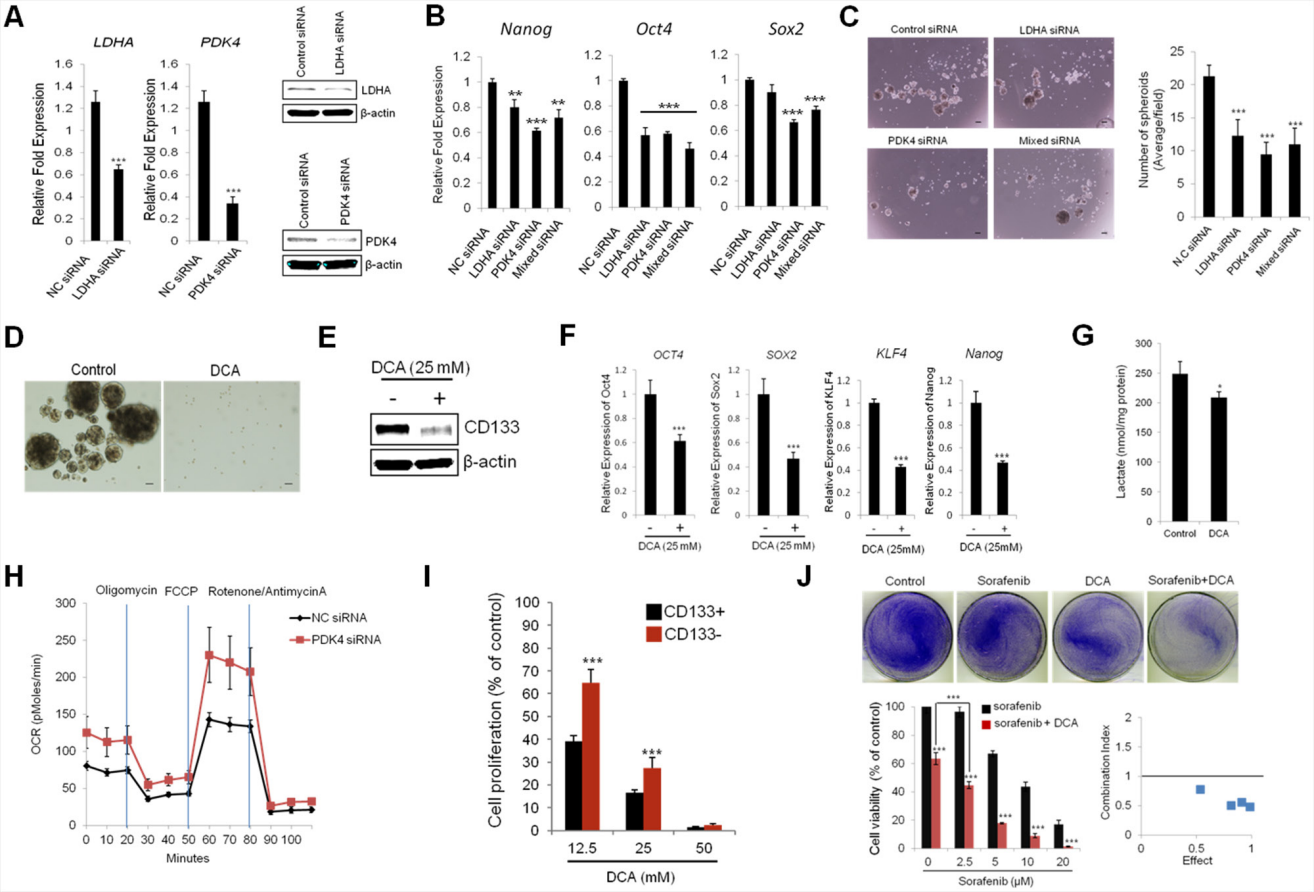
Targeting glycolytic enzymes inhibits stemness characteristics in CD133+ PLC/PRF/5 cells **A.** qRT-PCR and Western blotting analyses for LDHA and PDK4. **B.** Knockdown of LDHA and/or PDK4 inhibits stemness gene expression. CD133+ cells were transfected with siLDHA and/or siPDK4. The cells were incubated for 72 hrs and stemness genes expressions were determined by qRT-PCR. **C.** Knockdown of LDHA and/or PDK4 inhibits spheroid formation capacity. CD133+ cells were plated at low density on low attachment plate and the cells were transfected with siLDHA and/or siPDK4. 7 days after transfection the images were obtained and the numbers of spheroids were counted. **D.** Dichloroacetate (DCA) inhibits spheroid formation in CD133+ cells. The cells were plated at low density (5,000 cells/well) on low attachment plates and treated with DCA (12.5 mM). Spheroid formation was imaged after 7 days. **E.** DCA inhibits CD133 expression levels in CD133+ cells. The cells were treated with DCA (25 mM) for 72 hrs. CD133 expression levels were determined by Western blotting. **F.** DCA inhibits stemness gene expression. The CD133+ cells were treated with DCA (25 mM) for 72 hrs and qRT-PCR were performed to measure stemness gene expression. **G.** DCA inhibits lactate production in CD133+ cells. The cells were treated with DCA (25 mM) for 72 hrs. The cell culture supernatants were collected to determine lactate levels as described in Material and Methods. **H.** PDK4 depletion increases OCR in CD133+ cells. The cells transfected with PDK4 siRNA or scrambled siRNA were incubated for 48 hrs and OCR was measured after sequential incubation with 2 μM oligomycin, 2 μM FCCP and 0.5 μM Rotenone/antimycin A. **I.** CD133+ cells are more sensitive to DCA than CD133− cells. The cells were treated with indicated concentration of DCA for 72 hrs and the cell number was counted under a microscope. **J.** DCA sensitizes sorafenib-induced cell death. *Top panel*; CD133+ cells were treated with 5 μM sorafenib, 12.5 mM DCA, or in combination (sorafenib+DCA) for 48 hrs and the cells were stained with crystal violet. *Bottom panel;* CD133+ cells were treated with 12.5 mM DCA and various concentrations of sorafenib (0 - 20 μM) for 48 hrs and cell viability was determined by cell counting under the microscope. Combination index (CI) of DCA and sorafenib in CD133+ cells were calculated from the cell viability assay. All experiments were performed at least three times independently and the data shown are mean ± S.D. from three technical replicates of the experiments. **p < 0.01 and ***p < 0.001 (two-tailed Student's *t*-test). NC siRNA - negative control siRNA.

To investigate the impact of high glycolytic properties of CD133 (+) CSCs on stemness characteristics, CD133 (+) cells were transfected with siRNAs targeting lactate dehydrogenase A (LDHA), pyruvate dehydrogenase kinase 4 (PDK4), or both (mixed siRNA). The efficiency of siRNA-mediated knockdown was confirmed by qRT-PCR and Western blotting (Figure 3A). As shown in Figure 3B, knockdown of LDHA and PDK4 significantly decreased the expression of stemness genes (Nanog, Oct4 and Sox2) in CD133 (+) cells. The spheroid forming efficiency was markedly reduced by knockdown of LDHA and/or PDK4 (Figure 3C). In parallel, we also examined the effect of dichloroacetate (DCA), a pharmacological inhibitor of PDK, on stemness characteristics. As shown in Figure 3D, treatment of DCA significantly reduced the spheroid formation capacity of CD133 (+) cells. DCA treatment also decreased the expression of CD133 and stemness genes (Figure 3E and 3F). Reduced lactate production was confirmed in DCA-treated cells (Figure 3G). To determine metabolic shift from glycolysis to mitochondrial respiration, we measured oxygen consumption after PDK4 knockdown; our data showed that siRNA knockdown of PDK4 increased basal and maximal oxygen consumption rate (Figure 3H). These results support the notion that active glycolysis in CD133 (+) cells contribute to their stemness characteristics. As the CD133 (+) cells are highly glycolytic, we sought to further examine whether they are more sensitive to DCA treatment than the CD133 (−) cells. For this approach, the CD133 (+) and (−) cells were treated with various concentration of DCA (12.5 – 50 mM) for 72 hrs and the numbers of viable cells were counted. As shown in Figure 3I, although DCA decreased the proliferation of both CD133 (+) and CD133 (−) cells, the CD133 (+) cells were more sensitive to DCA treatment (growth inhibition rate 62% vs 37% at 12.5 mM DCA, respectively). Thus, the CD133 (+) cells appear to be sensitive to PDK inhibition, even though they are relatively resistant to sorafenib. We next examined the combination effect of DCA and sorafenib in CD133 (+) cells. As shown in Figure 3J, CD133 (+) cells treated with sorafenib in combination with DCA showed marked reduction of cell viability and the effect was greater than either DCA or sorafenib treatment alone; the combination index (CI) value ranged from 0.47 to 0.78. Therefore, glycolytic reprogramming can effectively inhibit CD133 (+) cell proliferation and their stemness characteristics, thus restoring sorafenib sensitivity.

### Extracellular glucose and lactate increase CD133 (+) cell population and their stemness characteristics

**Figure 4:**
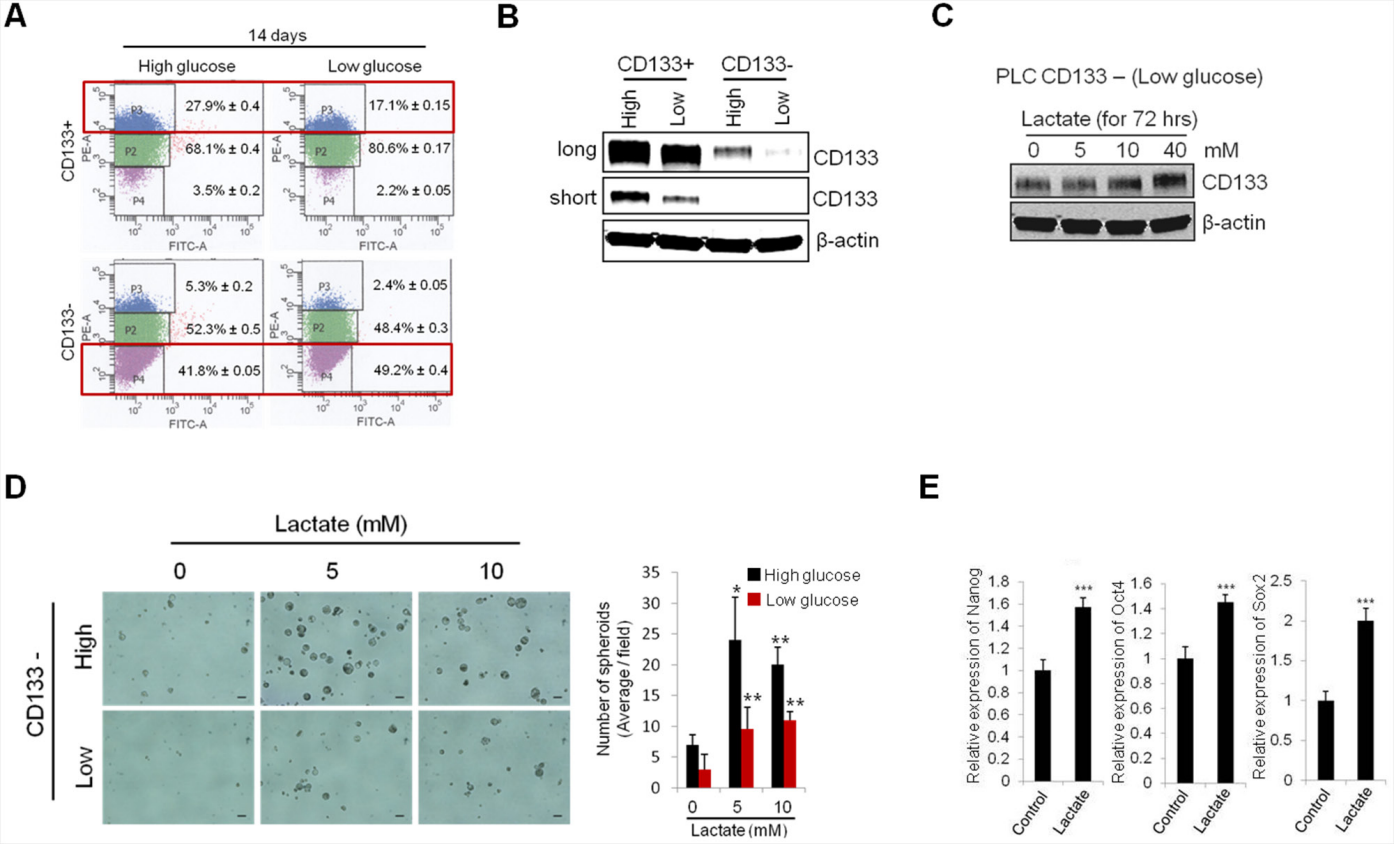
Extracellular glucose and lactic acid alters CD133 expression and stemness characteristics **A.** CD133+ and CD133− PLC/PRF/5 cells were cultured in medium containing high glucose and low glucose. After 14 days culture, CD133 positive and negative populations were measured by FACS analysis. **B.** Extracellular glucose levels affect the CD133 expression. After 13 days culture under either high or low glucose conditions, CD133 expression levels were measured by Western blotting. **C.** Treatment of lactate increased CD133 expression in CD133− PLC/PRF/5 cells. The cells were treated with various concentration of lactate for 72 hrs and the levels of CD133 were measured by Western blotting. **D.** Lactate treatment increased spheroid formation of CD133− cells. The cells were plated at low density on low attachment plate and treated with indicated concentrations of lactate. Represent images were obtained and counted to determined the numbers of spheroids after 9 days. **E.** CD133− cells were treated with lactate (5 mM) for 72 hrs and the stemness gene expressions were measured by qRT-PCR. All experiments were performed at least three times independently and the data shown are mean ± S.D. from three technical replicates of the experiments. *p < 0.05, **p < 0.01 and ***p < 0.001 (two-tailed Student's *t*-test).

Given that inhibition of glycolysis results in decreased CD133 expression and stemness characteristics in CD133 (+) cells, we next examined the effect of extracellular glucose concentration on CD133 (+) CSCs. For this purpose, the sorted CD133 (+) and CD133 (−) cells were cultured under high glucose (25 mM) or low glucose (5.5 mM) condition for 14 days and the subpopulation were measured by FACS analysis. We observed that the concentration of extracellular glucose in culture influenced the conversion between CD133 (+) and CD133 (−) cell populations (Figure 4A). For sorted CD133 (+) cells, the percentage of CD133 brightly stained population (P3 region) was significantly higher under high glucose condition compared to low glucose (27.9% vs 17.1%). For sorted CD133 (−) cells, the CD133 dimly stained population (P4 region) were more effectively maintained under low glucose concentration compared to high glucose (49.2% vs 41.8%). Western blotting analysis also showed decreased CD133 expression under low glucose conditions (Figure 4B). These results suggest that the concentrations of extracellular glucose influence the levels of CD133 expression.

Lactate is the end product of aerobic glycolysis; recently its role in tumor growth and progression has emerged [26]. We next examined whether lactate might regulate CD133 expression and stemness characteristics of CD133 (+) cells in our system. As shown in Figure 4C, exogenous lactate treatment significantly increased CD133 protein levels in CD133 (−) cells. The spheroid formation efficiency of CD133 (−) cells was markedly increased by lactate treatment (Figure 4D). Exogenous lactate treatment also increased the expression of stemness genes (Nanog, Oct4 and Sox2) (Figure 4E). Increased stemness gene expression and spheroid formation capacity was observed at relatively low concentration (5 mM) of lactate, although this lactate concentration did not increase CD133 protein level. The above results suggest that the levels of extracellular glucose and lactate are important environmental factors that influence the maintenance of CSCs populations.

### miR-122 inhibits the cell growth, glycolysis and spheroid formation by targeting PDK4 in CD133+ cells

**Figure 5:**
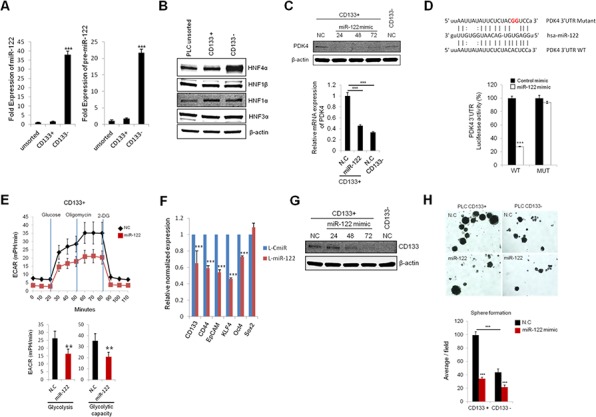
miR-122 inhibits glycolysis and spheroid formation by targeting PDK4 **A.** qRT-PCR for mature and precursor miR-122 expression in CD133+ and CD133− in CD133+ PLC/PRF/5 cells. The expression of miR-122 was significantly lower in CD133+ cells compared to CD133− cells. The data were normalized to U6 RNA. **B.** Western blotting for hepatic nuclear factors (HNFs) in unsorted, CD133+ and CD133− PLC/PRF/5 cells. CD133+ cells showed significantly lower expression of HNF4α than CD133− cells. **C.** Transient transfection of miR-122 mimics significantly inhibits the protein and mRNA expression of PDK4 in CD133+ PLC/PRF/5 cells. **D.** Luciferase activity in CD133+ cells transfected with plasmid encoding wild type (WT) or mutant (MUT) PDK4 3′UTR and treated with miR-122 or negative control miRNA. **E.** Real-time measurement of extracellular acidification rate (ECAR) showed that miR-122 inhibits glycolysis and glycolytic capacity in CD133+ cells. **F.** The effect of miR-122 on stem-cell associated gene expression in CD133+ PLC/PRF/5 cells. RNA was isolated from lenti-CmiR or lenti-pre-miR-122 infected cells and qRT-PCR was performed to determine the expression of indicated genes. **G.** The effect of miR-122 on CD133 expression levels. CD133+ PLC/PRF/5 cells were transfected with miR-122 mimic and incubated for indicated time periods. The cell lysates were then obtained for Western blotting analysis. **H.** The effect of miR-122 on spheroid formation of CD133+ PLC/PRF/5 cells. The cells were transiently transfected with miR-122 mimic; 24 hrs after transfection the cells were placed in low attachment plates. Spheroid formation was imaged after 7 days; the average numbers of spheroids are presented in the bar graph in the lower panel. All experiments were performed at least three times independently and the data shown are mean ± S.D. from three technical replicates of the experiments. *p < 0.05, **p < 0.01 and ***p < 0.001 (two-tailed Student's *t*-test). NC stands for negative control miRNA.

MiR-122, the liver specific miRNA, has been known to regulate cellular metabolism including lipid and cholesterol metabolism [27]; it is a pivotal tumor suppressor in the liver. However, to date, it remains unknown whether miR-122 is implicated in regulation of hepatocellular cancer stem cells. In the current study, we measured the levels of miR-122 in CD133 (+) and CD133 (−) cells. As shown in Figure 5A, the levels of mature and precursor miR-122 were significantly down-regulated in CD133 (+) CSCs. Given that the expression of miR-122 is known to be regulated by transcriptional activation through hepatic enriched transcription factors including hepatic nuclear factors (HNFs) [28], we examined the expression of HNFs in CD133 (+) and CD133 (−) cells. As shown in Figure 5B, the expression of HNF4α was evidently down-regulated in CD133 (+) CSCs. This finding suggests the possibility that HNF4α down-regulation may be responsible for reduction of miR-122 in CD133 (+) CSCs.

Our further experiments demonstrate that PDK4 is a direct target of miR-122. As shown in Figure 5C, the protein and mRNA levels of PDK4 were decreased by transient transfection of miR-122 mimic. Accordingly, we observed that the PDK4 3′UTR luciferase reporter activity was reduced by miR-122 mimic and that this effect was abolished by mutation of PDK4 3′UTR (Figure 5D). ECAR measurements showed that miR-122 mimic decreased glycolysis and glycolytic capacity in CD133 (+) cells (the non-glycolytic acidification level was relatively low) (Figure 5E).

**Figure 6:**
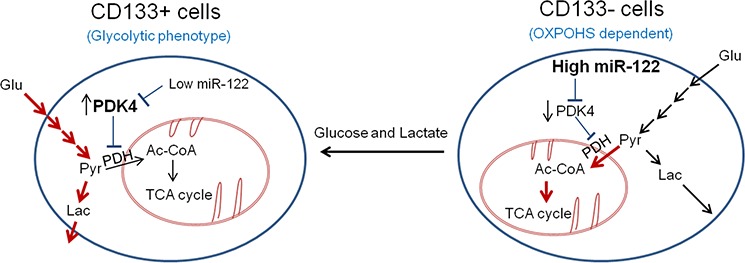
Schematic diagram of metabolic difference and regulation in CD133 + and CD133 − cells The CD133+ cells show glycolytic phenotype, while the CD133− cells show increased mitochondrial oxidative phosphorylation. The distinct metabolic phenotypes of CD133+ cells and CD133− cells are regulated by miR-122 and its direct target, PDK4. Whereas the CD133+ cells show low level of miR-122 which leads to increased expression of PDK4, the CD133− cells have high level of miR-122 which leads to reduced expression of PDK4. PDK4 is known to suppress mitochondrial OXPHOS through inactivation of PDH, leading to preferential use of pyruvate and generation of lactate. Our findings in this study demonstrate that MiR-122 mimic inhibits glycolysis and spheroid formation by targeting PDK4. Additionally, we show that extracellular glucose and lactic acid can alter CD133 expression and stemness characteristics. Thus, glycolytic phenotypes regulated by miR-122/PDK4 may play an important role in the stemness characteristic of CD133 (+) CSCs. Red arrows indicated active metabolic pathway in specific cell types.

Stable transduction of lentiviral vector containing primary miR-122 transcripts led to reduction of stemness gene (KLF4 and Oct4) and surface marker (CD133, CD44 and EpCAM) in CD133 (+) CSCs (Figure 5F). Reduction of CD133 expression was also confirmed by transfection with miR-122 mimic (Figure 5G). Furthermore, miR-122 mimic decreased the spheroid formation efficiency of in both CD133 (+) and CD133 (−) cells (Figure 5H). These results demonstrate that miR-122 inhibits CD133 (+) CSCs, at least in part, through glycolytic reprogramming via targeting PDK4 (Figure 6)

## DISCUSSION

Targeting cancer stem cells (CSCs) is an important emerging field in cancer biology because of the high tumorigenic capacity of CSCs and their resistance to chemo- and radiation therapy. Recent studies have identified several cancer stem-cell surface markers including CD133, CD44 and EpCAM, although global markers for CSCs in different human cancers are difficult to establish. CD133 has been one of the most frequently used surface markers for isolation of CSCs in several cancers [19]. Consistent with previous studies, we have found that CD133 (+) hepatocellular cancer cells highly express stemness genes (CD44, EpCAM, Oct4 and KLF4) and exhibit increased sphere formation capacity compared to the CD133 (−) cells. We show that the CD133 (+) cells are relatively resistant to sorafenib.

Abnormal metabolic phenotypes of cancer cell, such as aerobic glycolysis, have been considered as potential therapeutic targets for cancer treatments [29]. However, metabolic properties of CSCs still remain unclear. Recently, several studies have shown that CSCs predominantly perform glycolysis accompanied by increased glycolytic genes expression or decreased expression of genes that are involved in mitochondrial oxidative phosphorylation [12, 13, 30, 31]. However, some studies have reported opposite data that CSCs rely mainly on oxidative phosphorylation rather than glycolysis [14, 32]. These seemingly conflicting findings may be related, in part, to the different sources of CSCs and the methods used for isolation of CSCs. The current study provides the first evidence for distinct metabolic properties of CD133 (+) liver CSCs and their functional implication in the maintenance of stemness characteristics. We show that the CD133 (+) liver CSCs are characterized by increased expression of glycolytic genes (Glut1, HK2, PDK4 and PGAM1), high extracellular acidification rate (ECAR) and decreased expression of gluconeogenetic genes (G6Pase and Pepck). The CD133 (+) cells were found to have decreased cellular ATP levels with less mitochondrial respiration. All of these findings demonstrate that the CD133 (+) liver CSCs cells are more glycolytic than CD133 (−) cells. Previous studies have shown that CD133 (+) CSCs are predominantly distributed in the hypoxic niche [33] and their self-renewal capacity is increased under hypoxic conditions in several cancers [34–36]. The current study suggests an important role of glycolysis for the maintenance of stemness characteristics, as highlighted by the observations that knockdown (PDK4 and LDHA) or pharmacological inhibition (PDK) of glycolytic genes in CD133 (+) liver CSCs inhibited stemness gene expression and reduced spheroid formation. These findings suggest that aerobic glycolysis may play an important role in promoting cancer stem cell phenotype.

Recently, multiple therapeutic strategies have been proposed to eradicate CSCs population [37, 38]. For example, antibodies against CSCs surface marker such as CD133 or conjugated antibodies with cytotoxic agents have been reported to inhibit the self-renewal and tumorigenic capacity of CD133 (+) CSCs [39, 40]. In addition, targeting several embryonic signaling pathways (Notch, Hedgehog and Wnt/β-catenin) has also been proposed to eliminate CSCs, because of their deregulation in CSCs [41–44]. The current study shows that the CD133 (+) liver CSCs are more sensitive to DCA compared to the CD133 (−) cells. Our data indicate that combined treatment of DCA and sorafenib synergistically inhibits the growth of CD133 (+) cells. These results suggest that targeting aerobic glycolysis may preferentially eradicate the CD133 (+) liver CSCs and thus improve the response to chemotherapeutic agents. Given that aerobic glycolysis has been recognized as a hallmark of cancer cells [45], our data in the current study support that inhibition of aerobic glycolysis may be an effective therapeutic strategy by targeting both CSCs and bulk of tumor cells.

Recent studies have suggested that CSCs can be converted to non-CSCs, or vice versa; these processes are reversible and modulated by various environmental and cellular factors [46]. In our system, we have shown that the CD133 (+) and CD133 (−) cells are inter-convertible in culture and the processes are regulated by glucose concentrations (high concentration of glucose leads to more CD133+ cells; low concentration of glucose leads to more CD133− cells). Our findings suggest that high aerobic glycolysis in association with high glucose concentration favors the maintenance of CD133 (+) CSCs, while low aerobic glycolysis in association with low glucose causes reduction of CD133 (+) CSCs. Consistent with the documented role of lactate dehydrogenase-A (LDHA) for CSCs survival and proliferation as well as tumor progression [47], we observed that knockdown of LDHA decreased the stemness characteristics of the CD133 (+) cells. While lactate produced by LDHA had traditionally been considered as the waste product of glycolysis, recently it has been recognized as a critical regulator that affects cancer progression [26]. High glycolytic cells export lactate into the extracellular fluid, which is then imported by neighboring cancer cells or stoma cell through mono-carboxylic acid transporters (MCTs) [48]. Such a lactate shuttling results in metabolic reprogramming and also activation of several signaling pathways such as HIF1α and NF-kB [49, 50]. In the current study, we observed that lactate enabled the CD133 (−) cells to acquire stemness characteristics (reflected by increased spheroid formation and stemness gene expression). It is possible that lactate may enhance CD133 expression and stemness gene expression through generation of acetyl-coA, which can serve as acetyl-donor to influence histone acetylation, thus directly or indirectly regulating the expression of CD133 or other stemness genes. Our findings support a functional role of extracellular glucose and lactate as critical environmental factors for promoting/maintaining CSCs phenotypes.

Another important aspect of the current study relates to the novel role of the liver-specific miR-122 in regulation of glycolytic phenotypes and stemness characteristics of CD133 (+) CSCs. The significance of this part of the study is underscored by the fact that miR-122 is frequently decreased in human HCCs and that mice with miR-122 knockout developed hepatocellular cancer [17, 51]. Recently, miR-122 has been found to promote hepatic differentiation and maturation of embryonic stem cells (ESCs) by regulating the balance between proliferation and differentiation through miR-122/FoxA1/HNF4α positive feedback loop [52]. Interestingly, our current data showed that the expression of miR-122 was markedly decreased in CD133 (+) CSCs, which is consistent with the notion that CD133 (+) CSCs are relatively undifferentiated state of cells compared to CD133 (−) cells. Our functional studies demonstrate that overexpression of miR-122 in CD133 (+) cells inhibits aerobic glycolysis by directly targeting PDK4 and this effect leads to decreased expression of stemness genes and reduced spheroid formation. While several glycolytic inhibitors have been evaluated for clinical use, systemic toxicity hinder their clinical use for anti-cancer therapy [29]. Given that the expression of miR-122 is decreased in HCCs from patients with non-hepatitis C virus (non-HCV) infection [53] and that miR-122 inhibits aerobic glycolysis and CSCs phenotypes as shown in the current study, it is conceivable that miR-122 replacement therapy may represent a potential therapeutic strategy for the treatment of non-HCV-associated HCC; this approach is unlikely to be associated with significant side effect, as miR-122 is an endogenous molecule that is normally highly expressed in adult liver.

In our system, we observed that the expression of Sox2 was lower in CD133+ cells compared to CD133− cells. Although all pluripotent transcription factors including Sox2, Klf4, Nanog and Oct4 are implicated in the regulation of stemness, the levels of each transcription factor can be differentially regulated by multiple factors including genetic, epigenetic modification and miRNAs. Moreover, the levels of each transcription factors might be different depend on their differentiation status. For example, the expression pattern of Nanog and Oct4 is different from Sox2 in differentiating human embryonic stem cells [54]. Also, CSCs are known to exhibit heterogeneity and their differentiation status may vary depending on their cell types or intrinsic/extrinsic factors (such as genetic instability and environmental factors). Therefore, it is conceivable the expression of pluripotent transcription factors may vary depending on specific contexts.

In summary, the current study describes a distinct metabolic phenotype of CD133 (+) liver CSCs and an important role of glucose/lactate in promoting their stemness characteristics. Our findings suggest that targeting the glycolytic pathway may represent a promising therapeutic strategy to eradicate liver CSCs and to overcome therapeutic resistance. Moreover, our findings provide novel evidence for an important role of miR-122 in regulation of glycolysis and stemness characteristics of CD133 (+) CSCs.

## MATERIALS AND METHODS

### Cell culture, reagents and transfection

PLC/PRF/5, human hepatoma cell line was obtained from the American Type Culture Collection (Rockville, MD). The cells were cultured in low glucose (5.5 mM) or high glucose (25 mM) Dulbecco's modified Eagle's medium (DMEM) containing 10% fetal bovine serum (FBS; Gibco) and antibiotic. Mito Tracker Green and Mito Tracker red CMXRos were obtained from Invitrogen. Sodium lactate and sodium dichloroacetate were obtained from Sigma-Aldrich. The antibodies obtained were: anti-CD133/1 (AC133)-PE and anti-CD133 (W6B3C1) pure (Miltenyi Biotec), anti-HNF4α (Santa Cruz Biotechnology), anti-HNF1α and anti-HNF3α (Millipore), anti-HNF1β (Genway), anti-PDK4 (ABGENT), and anti-β-actin (Sigma). Synthetic siRNAs against LDHA and PDK4, miR-122, U6 primers and mimic were obtained from Qiagen. Cells were transfected at a final concentration of 50 nM siRNA or 20 nM of miR-122 mimic using Oligofectamine reagent (Invitrogen) according to the manufacturer's instructions. The lentiviral particle expressing miR-122 and lentiviral negative control mimic were obtained from GenCopoiea.

### Cell sorting

PLC/PRF/5 cells were sorted by flow cytometry using PE-conjugated human CD133/1 antibody (clone AC133, Miltenyi Biotec). The cells were trypsinized and washed with FACS wash buffer (PBS supplemented with 1% FBS), then incubated with antibody (1:100 in FACS wash buffer) for 30 min at 4°C. The cells were washed three times with FACS wash buffer and the CD133 (+) and CD133 (−) cell subpopulation were collected using a FACSAria and Diva 6.1.3 software (BD Biosciences). The top 20% most brightly stained cells or the bottom 10% dimly stained cells were selected as positive and negative populations, respectively.

### RNA isolation and quantitative RT-PCR

Total RNA was isolated using Trizol (Invitrogen) following manufacturer's instructions, and 1 μg of RNA was reverse transcribed into cDNA using iScript Reverse Transcription Supermix (Bio-Rad). Quantitative reverse transcription polymerase chain reaction (qRT-PCR) was performed with an SsoAdvanced SYBR Green Supermix (Bio-Rad) using Bio-Rad CFX96 Real-time PCR detection system. U6 small nuclear RNA was used as reference gene for miRNA-122; β-actin was used as reference gene for others (glycolytic and stemness genes). The expression of each gene was determined by 2-ΔΔCT method using the CFX Manager software (Biorad). The primer sequences used in this study are listed in Table 1.

**Table 1:** Primer sequences of genes confirmed with qRT-PCR

Gene Name	Primer sequences
CD133	Forward Primer. 5′-ATGCTCTCAGCTCTCCCGC-3′
	Reverse Primer. 5′-TTCTGTCTGAGGCTGGCTTG-3′
CD44	Forward Primer. 5′-GCAAACACAACCTCTGGTCC-3′
	Reverse Primer. 5′-CCCACACCTTCTTCGACTGT-3′
EpCAM	Forward Primer. 5′-GCTCTGAGCGAGTGAGAACC-3′
	Reverse Primer. 5′-ACGCGTTGTGATCTCCTTCT-3′
OCT4	Forward Primer. 5′-GGTGGAGGAAGCTGACAACA-3′
	Reverse Primer. 5′-GTTCGCTTTCTCTTTCGGGC-3′
SOX2	Forward Primer. 5′-GGGGAAAGTAGTTTGCTGCC-3′
	Reverse Primer. 5′-CGCCGCCGATGATTGTTATT-3′
KLF4	Forward Primer. 5′-GGGAGAAGACACTGCGTCAA-3′
	Reverse Primer. 5′-GGAAGTCGCTTCATGTGGGA-3′
NANOG	Forward Primer. 5′-ATGCCTCACACGGAGACTGT-3′
	Reverse Primer. 5′-AAGTGGGTTGTTTGCCTTTG-3′
ACTIN	Forward Primer. 5′-TGGAATCCTGTGGCATCCATGAAAC-3′
	Reverse Primer. 5′-TAAAACGCAGCTCAGTAACAGTCCG-3′
GLUT1	Forward Primer. 5′-TCACTGTGCTCCTGGTTCTG-3′
	Reverse Primer. 5′-CCTGTGCTCCTGAGAGATCC-3′
HK2	Forward Primer. 5′-TAGGGCTTTGAGAGCACCTGT-3′
	Reverse Primer. 5′-CCACACCACTGTCACTTTG-3′
LDHA	Forward Primer. 5′-ACGTCAGCAAGAGGGAGAAA-3′
	Reverse Primer. 5′-CGCTTCCAATAACACGGTTT-3′
PDK1	Forward Primer. 5′-CACGCTGGGTAATGAGGATT-3′
	Reverse Primer. 5′-ACTGCATCTGTCCCGTAACC-3′
PDK4	Forward Primer. 5′-CCTTTGGCTGGTTTTGGTTA-3′
	Reverse Primer. 5′-CCTGCTTGGGATACACCAGT-3′
PGAM1	Forward Primer. 5′-CCAAGAATCCCTGGACTGAA-3′
	Reverse Primer. 5′-TCAGACTGTCCTGCGTTTTG-3′
GAPDH	Forward Primer. 5′-AGGGCTGCTTTTAACTCTGGT-3′
	Reverse Primer. 5′-CCCCACTTGATTTTGGAGGGA-3′
G6Pase	Forward Primer. 5′-GCAATGGGCACTGGTATTTG-3′
	Reverse Primer. 5′-TGGAGTCACACATGGGAATAAG-3′
PEPCK	Forward Primer. 5′-TAGCACCCTCATCTGGGAATA-3′
	Reverse Primer. 5′-GTCTTTGTGGGAAGGTCTATGG-3′

### Western blotting

Cells were lysed with RIPA buffer containing protease inhibitors cocktail (Roche). After sonication on ice, the cell lysates were centrifuged at 12,000 g for 15 min at 4°C and the supernatants were collected. The lysates were separated by SDS-PAGE and then transferred onto the nitrocellulose membrane (BioRad). Following blocking with 5% nonfat milk in PBS-T (PBS containing 0.1% Tween 20) for 1 hr, the membranes were incubated with antibodies against CD133, PDK4, HNF4α, HNF1α, HNF1β, HNF3α, β-actin at appropriate dilutions at 4°C overnight. Membranes were washed three times for 10 min and incubated with secondary antibody (IRDye conjugated IgG, LI-COR) in PBS-T containing 5% nonfat milk for 1 hr. The signals were then detected with Odyssey Imaging System (LI-COR).

### Construction of PDK4 3′UTR luciferase reporter plasmid and luciferase reporter activity assay

To construct a plasmid containing PDK4 3′-untranslated region (UTR), human PDK4 3′-UTR sequences were amplified using the specific primers: forward 5′-TCCAAAGGCATCCATTTTCT-3′ and reverse 5′-AAGCATGCTGGCATGAGAAT-3′. The amplified 200-bp fragment containing the miR-122 target site was cloned into the pMIR-REP-dCMV luciferase reporter vector with restriction enzyme Mlu1/HindIII insert. Mutation of the putative miR-122 target sites at the PDK4 3′UTR was achieved by using the QuikChange II site-directed mutagenesis kit (Agilent Technologies). The accuracy of the produced constructs was confirmed by DNA sequencing.

### Spheroid formation assay

The sorted cells were plated at a density of 1,000 cells/well in 24 well ultra low attachment plates (Corning) in serum-free DMEM/F12 supplemented with B-27 (50X), epidermal growth factor (EGF; 20 ng/ml) and fibroblast growth factor (FGF; 10 ng/ml). After 6–9 days incubation, spheroids images were obtained using light inverted microscope. The numbers of spheroids at different sizes (50–100 μm or > 100 μm) were counted under microscope (at least 5 wells per sample).

### Measurement of extracellular acidification and oxygen consumption rate

Extracellular Acidification Rate (ECAR) and Oxygen Consumption Rate (OCR) were measured using XF24 Extracellular Flux Analyzer (Seahorse Bioscience). For ECAR measurement, the cells (3.5 × 10^4^/well) were plated in XF24 cell-culture microplate; after 24 hrs the culture medium was replaced with XF assay medium containing 2 mM L-glutamine. ECAR was measured by sequential addition of glucose, oligomycin (mitochondrial H^+^-ATP-Synthase inhibitor, final concentration 2.5 μM), and 2-deoxyglucose (glycolysis inhibitor, final concentration 100 mM) in an XF24 flux analyzer. The glycolysis and glycolytic capacity were calculated: Glycolysis = ECAR after addition of glucose – ECAR after 2-DG treatment; Glycolytic capacity = ECAR after oligomycin treatment – ECAR after 2DG treatment. For OCR measurement, the cells (5 × 10^4^/well) were plated in XF24 cell-culture microplate; after 24 hrs the culture medium was replaced with the XF assay medium containing 1 mM pyruvate, 2 mM glutamine, and 10 mM glucose. OCR was measured by sequential addition of oligomycin (final concentration 2 μM), FCCP (final concentration 2 μM), and Rotenone/antimycin A (final concentration 0.5 μM). At the end of each assay, the cells in each well were trypsinized and counted; the Seahorse data were normalized to relative cell numbers of each well.

### Measurement of ATP and lactate

Cellular ATP contents were measured by using an ATPlite assay kit according to the manufacturer's protocol (Perkin-Elmer Life Sciences). Briefly, 100 μl of the cell lysate were mixed with 100 μl of substrate solution and the plates were shaken for 2 min at 700 rpm using orbital plate shaker. The plates were then incubated for 10 min. Luminescence was measured by using a luminescence analyzer (Berthold LB960). Lactate was measured by using Lactate assay kit according to the manufacturer's protocol (Sigma-aldrich). Specifically, the cell culture supernatant was collected and deproteinized with a 10 kDa MWCO spin filter. The samples were then diluted using lactate assay buffer and mixed with reaction mixure (lactate probe, lactate enzyme mix), followed by incubation at room temperature for 30 min and subsequent measurement for absorbance at 570 nm.

### Statistics

Statistical analyses were performed using Graphpad Prism. The statistical significance was determined with a two-tailed Student's *t*-test for unpaired data. *P* values are indicated with *p < 0.05, **p < 0.01, ***p < 0.001. Combination index (CI) value was calculated by Chou-Talalay method using CompuSyn software (Combosyn, Inc, Paramus, NJ) (CI < 1, synergy; CI = 1, additive effect; CI > 1, antagonism).
